# The weekend effect on the provision of Emergency Surgery before and during the COVID-19 pandemic: case–control analysis of a retrospective multicentre database

**DOI:** 10.1186/s13017-022-00425-z

**Published:** 2022-04-29

**Authors:** Giovanni D. Tebala, Marika S. Milani, Roberto Cirocchi, Mark Bignell, Giles Bond-Smith, Christopher Lewis, Vanni Agnoletti, Marco Catarci, Salomone Di Saverio, Gianluigi Luridiana, Fausto Catena, Marco Scatizzi, Pierluigi Marini, the CovidICE-International Collaborative, R. Lo Dico, A. Stracqualursi, G. Russo, S. D’Errico, P. Cianci, E. Restini, G. Scialandrone, G. Guercioni, G. Martinez, A. Pezzolla, D. F. Altomare, A. Picciariello, G. Trigiante, R. Dibra, V. Papagni, C. Righetti, R. Polastri, J. Andreuccetti, G. Pignata, R. D’Alessio, E. Arici, I. Canfora, N. Cillara, A. Deserra, R. Sechi, F. Bianco, S. Gili, A. Cappiello, P. Incollingo, A. Biloslavo, G. Bellio, P. Germani, N. De Manzini, M. Buiatti, F. P. Paladino, D. Sasia, F. Borghi, V. Testa, G. Giraudo, F. Allisiardi, M. C. Giuffrida, M. Gerosa, A. Fogliati, D. Maggioni, N. Fabbri, C. V. Feo, E. Bianchini, I. Panzini, V. Lizzi, F. G. Tricarico, G. Di Gioia, R. Melino, N. Tartaglia, A. Ambrosi, G. Pavone, M. Pacilli, F. Vovola, F. Belli, A. Barberis, A. Azzinnaro, A. Coratti, R. Benigni, S. Berti, M. Saracco, A. Gennai, L. Dova, R. Farfaglia, G. Pata, V. Arizzi, G. Pandolfo, A. Frontali, P. Danelli, L. Ferrario, C. Guerci, N. M. Mariani, A. Pisani Ceretti, V. Nicastro, E. Opocher, D. Gozzo, G. Casoni Pattacini, M. Castriconi, A. Amendola, M. Gaudiello, G. Palomba, F. Catena, G. L. Petracca, G. Perrone, M. Giuffrida, G. Moretto, H. Impellizzeri, A. Casaril, M. Filosa, A. Caizzone, S. Agrusti, G. M. Cattaneo, P. Capelli, A. Muratore, M. Calabrò, N. Pipitore Federico, B. Cuzzola, R. Danna, A. Murgese, F. Coccolini, E. Pieroni, M. Chiarugi, D. Tartaglia, S. Giannessi, R. Somigli, M. Trafeli, M. Fedi, R. De Vincenti, A. Guariniello, M. Grande, G. Bagaglini, B. Pirozzi, A. M. Guida, S. Ingallinella, C. P. Don, L. Siragusa, O. Capone, D. Cerbo, E. Santoro, V. Pende, A. Fassari, A. Mingoli, G. Brachini, B. Cirillo, M. Zambon, P. Cicerchia, S. Meneghini, P. Sapienza, A. Puzzovio, F. La Torre, P. Fransvea, M. Di Grezia, G. Sganga, M. F. Armellino, G. Ioia, B. Rampone, M. Della Corte, F. Fleres, G. Clarizia, P. Bordoni, A. Spolini, M. Franzini, A. Grechi, M. Suppo, D. Bono, D. Scaglione, C. Cotsoglou, S. Paleini, A. P. Chierici, M. Uccelli, S. Olmi, G. Cesana, N. Tenreiro, A. Marcal, D. Martins, C. Leal, B. Vieira, B. Ugarte-Sierra, I. Vincene-Rodriguez, M. Duran-Ballesteros, A. Sanz-Larrainzar, F. J. Ibanez-Aguirre, C. Yanez-Benites, I. Talal, J. L. Blas, R. Garau, S. Clark-Stuart, A. Wallace, A. Di Carlo, E. Wisnia, K. Ehsan, K. Beck-Sanders, E. Godson, P. Campbell, G. D. Tebala, M. Bignell, G. Bond-Smith, C. Lewis, R. Ahmad, R. Ali, S. S. Aswani, A. Barza, C. Carrillo, A. Dawani, A. Dey, A. Elserafy, D. Gaspar, L. Lazzareschi, M. Patel, A. Shabana, M. Shams, O. Shams, Z. Slack

**Affiliations:** 1 Surgical Emergency Unit, Oxford University Hospitals NHS Foundation Trust, Oxford, UK; 2 Digestive and Emergency Surgery Unit, Azienda Ospedaliera “S.Maria”, “S.Maria” Hospital, Viale Tristano di Joannuccio, 05100 Terni, Italy; 3 Department of General Surgery, Causa Pia Luvini Hospital, Cittiglio, Italy; 4 Department of Anaesthesia and Intensive Care, “M. Bufalini” Hospital, Cesena, Italy; 5 Department of General Surgery, “S. Pertini” Hospital, Rome, Italy; 6 Department of General Surgery, Madonna del Soccorso Hospital, S.Benedetto del Tronto, Italy; 7 Department of Oncologic Surgery, “A. Businco” Hospital, Cagliari, Italy; 8 Department of General and Emergency Surgery, “M. Bufalini” Hospital, Cesena, Italy; 9 Department of General Surgery, S.Maria Annunziata Hospital, Florence, Italy; 10 Department of General and Emergency Surgery, S.Camillo-Forlanini Hospital, Rome, Italy

**Keywords:** Weekend effect, Emergency surgery, Hot gallbladder

## Abstract

**Introduction:**

The concept of “weekend effect”, that is, substandard healthcare during weekends, has never been fully demonstrated, and the different outcomes of emergency surgical patients admitted during weekends may be due to different conditions at admission and/or different therapeutic approaches. Aim of this international audit was to identify any change of pattern of emergency surgical admissions and treatments during weekends. Furthermore, we aimed at investigating the impact of the COVID-19 pandemic on the alleged “weekend effect”.

**Methods:**

The database of the CovidICE-International Study was interrogated, and 6263 patients were selected for analysis. Non-trauma, 18+ yo patients admitted to 45 emergency surgery units in Europe in the months of March–April 2019 and March–April 2020 were included. Demographic and clinical data were anonymised by the referring centre and centrally collected and analysed with a statistical package. This study was endorsed by the Association of Italian Hospital Surgeons (ACOI) and the World Society of Emergency Surgery (WSES).

**Results:**

Three-quarters of patients have been admitted during workdays and only 25.7% during weekends. There was no difference in the distribution of gender, age, ASA class and diagnosis during weekends with respect to workdays. The first wave of the COVID pandemic caused a one-third reduction of emergency surgical admission both during workdays and weekends but did not change the relation between workdays and weekends. The treatment was more often surgical for patients admitted during weekends, with no difference between 2019 and 2020, and procedures were more often performed by open surgery. However, patients admitted during weekends had a threefold increased risk of laparoscopy-to-laparotomy conversion (1% vs. 3.4%). Hospital stay was longer in patients admitted during weekends, but those patients had a lower risk of readmission. There was no difference of the rate of rescue surgery between weekends and workdays. Subgroup analysis revealed that interventional procedures for hot gallbladder were less frequently performed on patients admitted during weekends.

**Conclusions:**

Our analysis revealed that demographic and clinical profiles of patients admitted during weekends do not differ significantly from workdays, but the therapeutic strategy may be different probably due to lack of availability of services and skillsets during weekends. The first wave of the COVID-19 pandemic did not impact on this difference.

## Introduction

The so-called weekend effect is a suspected epidemiological effect representing a source of serious concern for healthcare professionals, policy-makers and the general public. It has been claimed that during weekends workload and capacity of healthcare systems may change completely with respect to workdays, causing substandard care and suboptimal results. The first definition of “weekend effect” was related to a reported increase of mortality in patients admitted during weekends, but nowadays it refers—more widely—to the differences in the provision of healthcare between workdays and weekend. In the present paper, the “weekend effect” will be considered in this wider sense.

In 2016, the then UK Secretary of State for Health claimed that 11,000 deaths per year could be caused by the “weekend effect”, raising concerns among the population and leading to the implementation of the “seven-day hospital services” policy in the NHS [1]. However, that statement was subsequently found to be inaccurate as the comparison between workdays and weekends did not take into consideration that patients admitted to Emergency Departments during weekends were usually more ill and in poorer general conditions. At that time, several academics wrote to the Secretary of State for Health to complain about that misrepresentation of facts based on “bad” evidence, only for political convenience. Nevertheless, the evidence behind an alleged weekend effect is still unclear. Moreover, the massive shake-up of our healthcare systems caused in the last 2 years by the COVID pandemic could have exacerbated an eventual weekend effect, due to an alleged further reduction of capacity during lockdown weekends, but this has not been demonstrated.

To confirm or rule out the impact of a “weekend effect” on emergency surgical admissions, we have interrogated our large international database on emergency surgical admission before and during the COVID pandemic.

## Materials and methods

Data for this study were derived from the CovidICE-International Study database, whose characteristics have been reported elsewhere [2].

The initial recruitment of participating units was done by emailing an invitation letter to more than 6000 surgeons in Europe. Forty-five Emergency Surgical units decided to contribute to the CovidICE-International database. A local team led by a Principal Local Investigator (PLI) for each centre collected anonymised demographic and clinical data of patients and transmitted them to the Principal Investigator (PI) and the Study Coordinator (SC) within an encrypted electronic database (MS Excel for Mac). Data were centrally collected, double-checked and analysed with a statistical package (StatPlus for Mac). Only 18+, non-trauma patients admitted for a surgical emergency during the months of March–April 2019 and March–April 2020 were analysed. The final database comprises data of 6263 completely anonymised patients.

Primary endpoint of the present analysis was to see whether there was any difference in admissions, diagnoses and treatments during the weekend (Saturday–Sunday) with respect to workdays. In particular, we evaluated if distribution of demographic and clinical variables (age, gender, ASA, frailty [3], diagnosis, primary treatment, surgical access, laparoscopy-to-laparotomy conversion rate, length of stay, rate of rescue surgery, rate of readmission) changed during weekends versus workdays. The category "hot gallbladder" includes acute cholecystitis and intractable biliary colic. The categories "pancreatitis" and "diverticulitis" include both complicated and non-complicated acute pancreatitis and acute diverticulitis, respectively.

Secondary endpoint was to see whether the so-called weekend effect changed during the COVID-19 pandemic with respect to the pre-pandemic period. The months of March and April 2020 were chosen as they represent the onset of the pandemic. They were compared with the same 2 months of 2019 to avoid seasonal bias.

Trauma patients, patients < 18 yo and those with more than 20% of incomplete data were excluded from the analysis. Factors with more than 10% of missing data were excluded from the analysis. Missing data were excluded listwise.

Frequency variables were analysed with the Pearson Chi-square test and with the 2-way ANalysis-Of-VAriance (ANOVA). Continuous variables were compared with the Mann–Whitney U test after a first distribution analysis confirmed non-normal distribution. Statistical significance was confirmed when *p* < 0.05.

Ethical committee approval was not deemed to be necessary as this study is a retrospective audit on completely anonymised data. The study was endorsed by the Association of Italian Hospital Surgeons (Associazione dei Chirurghi Ospedalieri Italiani—ACOI) and by the World Society of Emergency Surgery (WSES).

This paper has been drafted according to the STrengthening the Reporting of OBservational studies in Epidemiology (STROBE) checklist [4].

## Results

**Fig. 1:**
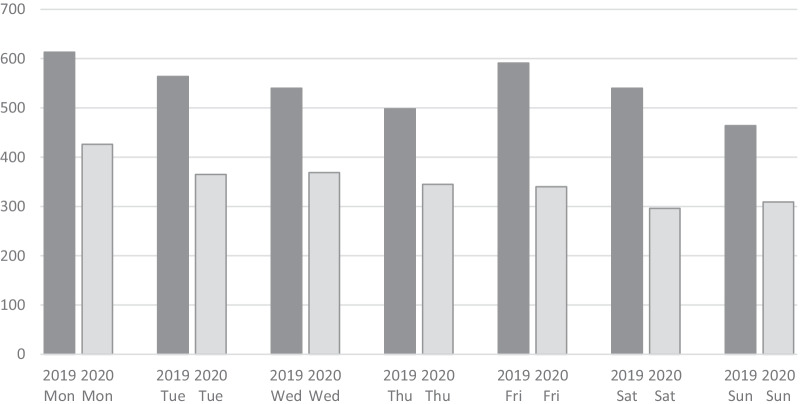
Admissions by period (March–April 2019 vs. March–April 2020) and day of the week

**Fig. 2:**
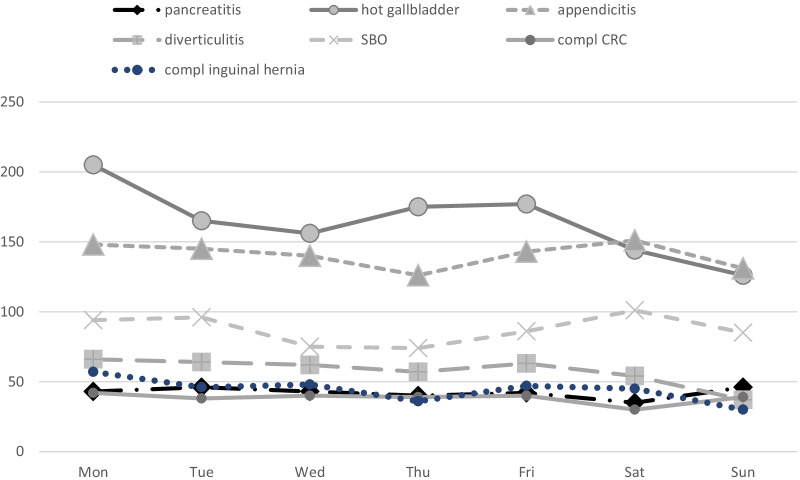
Admissions per day of the week, seven most common diagnoses. The category “hot gallbladder” includes acute cholecystitis and intractable biliary colic. *SBO* small bowel obstruction, *CRC* colorectal cancer

Results are summarised in Tables 1, 2, 3, 4, 5, 6, 7, 8, 9 and 10 and Figs. 1 and 2.

**Table 1:** Basic characteristics

	Workday (Monday–Friday)		Weekend (Saturday–Sunday)		*p*
Total	4654 (74.3%)		1609 (25.7%)		
Gender					
M	2380		797		0.267
F	2274		812		
Age	59.5 ± 20.8		59.2 ± 20.7		0.735
ASA					
1	1104		379		0.825
2	1483		519		
3	1307		446		
4	379		136		
5	21		11		
Frailty score					
1–2	2384		841		0.49235
> 2	2269		768		
*	**2019**	**2020**	**2019**	**2020**	
1–2	1504	880	556	285	
> 2	1302	967	448	320	
	*p* = 0.00008		*p* = 0.00116		

* Bold indicates distribution of Frailty Score in the two periods of the study

**Table 2:** Variation of the most frequent diagnoses

	Tot	Workday (Monday–Friday)		Weekend (Saturday–Sunday)		*p*
		2019	2020	2019	2020	
Hot gallbladder	1148	533	347	168	100	0.5334
Acute appendicitis	983	437	263	184	99	0.4461
SBO	611	266	159	113	73	0.6671
Diverticulitis	403	208	102	60	33	0.6434
Complicated inguinal hernia	309	147	87	52	23	0.3053
Pancreatitis	295	119	93	55	28	0.1116
Complicated CRC	268	117	82	37	32	0.4541

**Table 3:** Results

	Workday (Monday–Friday)		Weekend (Saturday–Sunday)		*p*
	2019	2020	2019	2020	
Primary treatment					
Medical	1489 (75.1%) (32.0%)		494 (24.9%) (30.7%)		0.037
Surgical	2861 (73.4%) (61.5%)		1035 (26.6%) (64.3%)		
IR/Endo	301 (79.0%) (6.5%)		80 (21.0%) (5.0%)		
Medical	875	614	300	194	0.441
Surgical	1744	1117	647	388	0.379
IR/Endo	187	114	57	23	0.131
Surgical access					
Laparos	1110 (73.5%) (42.0%)		401 (26.5%) (41.4%)		0.004
Open	1522 (73.3%) (57.6%)		553 (26.7%) (57.1%)		
Convert	11 (44.0%) (0.4%)		14 (56.0%) (1.4%)		
Laparos	696	414	256	145	0.686
Open	915	607	342	211	0.477
Converted	11	0	8	6	0.013
Conversion rate	11/1121 (1.0%)		14/415 (3.4%)		0.001
	11/707	0/414	8/264	6/151	
	*p* = 0.011		*p* = 0.609		
Length of stay	7.6 ± 9.9, 5 (0–231)^$^		8.1 ± 11.4, 5 (0–220)^$^		0.033^$^
	7.7 ± 10.7	7.4 ± 8.6	7.9 ± 10.0	8.6 ± 13.5	
	*p* = 0.664		*p* = 0.011		
Rescue surgery					
No	4323 (74.6%) (96.9%)		1468 (25.3%) (95.9%)		0.061
Yes	139 (68.8%) (3.1%)		63 (31.2%) (4.1%)		
	84	55	36	27	0.659
Readmission					
No	4182 (74.1%) (91.1%)		1463 (25.9%) (92.9%)		0.028
Yes	408 (78.5%) (8.9%)		112 (21.5%) (7.1%)		
	265	143	70	42	0.631

Data presented as absolute number and percentage within row and within column, respectively

^$^ = Data reported as mean ± standard deviation, median (range); comparison with Mann–Whitney U test

**Table 4:** Subgroup analysis. Pancreatitis

	Workday (Monday–Friday)	Weekend (Saturday–Sunday)	*p*
Primary treatment			
Medical	167 (78.8%)	61 (73.5%)	0.5052
Surgical	32 (15.1%)	14 (16.9%)	
IR/Endo	13 (6.1%)	8 (9.6%)	
Surgical access			
Laparos	23 (85.2%)	13 (86.7%)	0.8954
Open	4 (14.8%)	2 (13.3%)	
Converted	0	0	
Conversion rate	0	0	
Length of stay^$^	7.5 ± 8.9	8.8 ± 13.2	0.8797
	5 (0–82)	5 (1–79)	
Readmission			
No	160 (70.2%)	68 (84.0%)	0.1885
Yes	48 (29.8%)	13 (16.0%)	

Data presented as absolute number and percentage within column, respectively

^$^ = Data reported as mean ± standard deviation, median (range); comparison with Mann–Whitney U test

**Table 5:** Subgroup analysis. Hot gallbladder

	Workday (Monday–Friday)	Weekend (Saturday–Sunday)	*p*
Primary treatment			
Medical	252 (28.7%)	86 (32.1%)	0.0157
Surgical	490 (55.8%)	159 (59.3%)	
IR/Endo	136 (15.5%)	23 (8.6%)	
Surgical access			
Laparos	374 (80.9%)	113 (73.4%)	0.0774
Open	85 (18.4%)	38 (24.7%)	
Converted	3 (50.0%)	3 (50.0%)	
Conversion rate	3/377 (0.8%)	3/116 (2.6%)	0.124
Length of stay^$^	6.5 ± 6.6	7.3 ± 7.1	0.0228
	5 (0–67)	5 (0–60)	
Readmission			
No	765 (87.8%)	239 (90.5%)	0.2290
Yes	106 (12.2%)	25 (9.5%)	

Data presented as absolute number and percentage within column, respectively

^$^ = Data reported as mean ± standard deviation, median (range); comparison with Mann–Whitney U test

**Table 6:** Subgroup analysis. Appendicitis

	Workday (Monday–Friday)	Weekend (Saturday–Sunday)	*p*
Primary treatment			
Medical	58 (8.3%)	27 (9.5%)	0.8041
Surgical	640 (91.4%)	255 (90.1%)	
IR/Endo	2 (0.3%)	1 (0.4%)	
Surgical access			
Laparos	492 (80.8%)	198 (80.5%)	0.8606
Open	112 (18.4%)	45 (18.3%)	
Converted	5 (0.8%)	3 (1.2%)	
Conversion rate	5/497 (1.0%)	3/201 (1.5%)	0.5850
Length of stay^$^	4.0 ± 3.7	4.4 ± 4.6	0.1176
	3 (0–36)	3 (0–55)	
Readmission			
No	677 (97.4%)	272 (96.5%)	0.4170
Yes	18 (2.6%)	10 (3.5%)	

Data presented as absolute number and percentage within column, respectively

^$^ = Data reported as mean ± standard deviation, median (range); comparison with Mann–Whitney U test

**Table 7:** Subgroup analysis. Diverticulitis

	Workday (Monday–Friday)	Weekend (Saturday–Sunday)	*p*
Primary treatment			
Medical	184 (59.4%)	48 (51.6%)	0.3752
Surgical	115 (37.1%)	42 (45.2%)	
IR/Endo	11 (3.5%)	3 (3.2%)	
Surgical access			
Laparos	16 (14.3%)	9 (21.4%)	0.2843
Open	96 (85.7%)	33 (78.6%)	
Converted	0	0	
Conversion rate	0	0	
Length of stay^$^	10.0 ± 11.7	9.4 ± 7.6	0.4917
	7 (0–150)	7 (2–42)	
Readmission			
No	277 (91.4%)	81 (90.0%)	0.6781
Yes	26 (8.6%)	9 (10.0%)	

Data presented as absolute number and percentage within column, respectively

^$^ = Data reported as mean ± standard deviation, median (range); comparison with Mann–Whitney U test

**Table 8:** Subgroup analysis. Small bowel obstruction

	Workday (Monday–Friday)	Weekend (Saturday–Sunday)	*p*
Primary treatment			
Medical	170 (40.0%)	64 (34.4%)	0.3827
Surgical	249 (58.6%)	120 (64.5%)	
IR/Endo	6 (1.4%)	2 (1.1%)	
Surgical access			
Laparos	40 (17.0%)	23 (19.7%)	0.0235
Open	194 (82.6%)	89 (76.1%)	
Converted	1 (0.4%)	5 (4.3%)	
Conversion rate	1/41 (2.4%)	5/28 (17.9%)	0.046
Length of stay^$^	8.6 ± 7.6	8.1 ± 5.8	0.3542
	6.5 (0–58)	7 (0–31)	
Readmission			
No	396 (94.5%)	173 (94.0%)	0.8105
Yes	23 (5.5%)	11 (6.0%)	

Data presented as absolute number and percentage within column, respectively

^$^ = Data reported as mean ± standard deviation, median (range); comparison with Mann–Whitney U test

**Table 9:** Subgroup analysis. Complicated colorectal cancer

	Workday (Monday–Friday)	Weekend (Saturday–Sunday)	*p*
Primary treatment			
Medical	17 (8.5%)	3 (4.3%)	0.3757
Surgical	172 (86.4%)	64 (92.7%)	
IR/Endo	10 (5.0%)	2 (2.9%)	
Surgical access			
Laparos	24 (14.8%)	9 (15.3%)	0.0616
Open	138 (85.2%)	48 (81.4%)	
Converted	0	2 (3.4%)	
Conversion rate	0	2/11 (18.2%)	0.031
Length of stay^$^	13.7 ± 10.8	13.3 ± 14.3	0.3232
	11 (1–68)	11 (1–117)	
Readmission			
No	178 (89.9%)	62 (91.2%)	0.7596
Yes	20 (10.1%)	6 (8.8%)	

Data presented as absolute number and percentage within column, respectively

^$^ = Data reported as mean ± standard deviation, median (range); comparison with Mann–Whitney U test

**Table 10:** Subgroup analysis. Complicated inguinal hernia

	Workday (Monday–Friday)	Weekend (Saturday–Sunday)	*p*
Primary treatment			
Medical	17 (7.3%)	2 (2.7%)	0.1491
Surgical	217 (92.7%)	73 (97.3%)	
IR/Endo	0	0	
Surgical access			
Laparos	1 (0.5%)	3 (4.2%)	0.0235
Open	206 (99.5%)	69 (95.8%)	
Converted	0	0	
Conversion rate	0	0	
Length of stay^$^	4.4 ± 6.3	4.2 ± 4.2	0.1150
	2 (0–50)	3 (0–25)	
Readmission			
No	224 (96.1%)	72 (96.0%)	0.9574
Yes	9 (3.9%)	3 (4.0%)	

Data presented as absolute number and percentage within column, respectively

^$^ = Data reported as mean ± standard deviation, median (range); comparison with Mann–Whitney U test

Of the 6263 patients, 74.3% were admitted during the working days, while only 25.7% were admitted during the weekends.

Gender distribution, average age, ASA class and distribution of frailty score did not change during the weekend with respect to working days (Table 1). However, our data showed that the distribution of frailty score was significantly different between 2019 and 2020, both during weekdays and weekends (Table 1).

The general profile of admissions during the week is quite constant, but for each day of the week there is an evident difference in the number of admissions between March–April 2019 and March–April 2020. This different is pretty much constant for the whole week (Monday − 30.5%, Tuesday − 35.3%, Wednesday − 31.7%, Thursday − 30.7%, Friday − 42.5%, Saturday − 45.2%, Sunday − 33.4%) (Fig. 1). The average reduction of admissions between 2019 and 2020 was 34.2% during workdays and 39.7% during weekends (*p* = 0.143).

The seven most common diagnoses were: (1) hot gallbladder, (2) acute appendicitis, (3) small bowel obstruction (SBO), (4) diverticulitis, (5) complicated inguinal hernia, (6) pancreatitis, (7) complicated colorectal (CRC) cancer (Table 2). Their distribution did not show any significant difference between weekends and working days (Table 2), although the number of admitted hot gallbladders tends to be highest on Mondays and then progressively reduces to become lowest on Sundays (Fig. 2).

The treatment was more frequently surgical in the patients admitted during weekends (*p* = 0.037) with respect to workdays, but the distribution of the three types of treatments (medical, surgical or interventional) did not show any change between 2019 and 2020 (Table 3).

The surgical access (laparoscopic vs. open) did not change, but patients admitted during weekends experienced a threefold increased risk of laparoscopy-to-laparotomy conversion (1% vs. 3.4%, *p* = 0.001). The risk of conversion was lowest in patients admitted on workdays during the pandemic (Table 3).

Nonparametric comparison showed that hospital stay was longer in patients admitted during the weekend (*p* = 0.033) and, within this group, it was significantly longer during the COVID period (Table 3).

The rate of rescue surgery—defined as an operation performed due to failure of the primary treatment (medical, surgical or endoscopic/interventional)—was not statistically different in the two groups (workdays vs. weekends).

Readmission rate was lower in patients admitted during weekends (*p* = 0.028). No variation was found in the rate of readmission during weekend versus workdays between 2019 and 2020 (Table 3). An ad hoc regression analysis was performed to investigate the relation between risk of readmission and length of stay (LOS), and this confirmed the presence of a minimal but significant direct association (risk of readmission =  − 2.44862 + 0.00787 * LOS, *p* = 0.03762). This finding was confirmed at Pearson’s correlation analysis (*R* = 0.029, *p* = 0.0233).

Subgroup analysis has been performed on the seven most frequent diagnoses (Tables 4, 5, 6, 7, 8, 9, 10). Patients with hot gallbladder have been mostly treated with emergency surgery, but the percentage of those having an interventional procedure (cholecystostomy) almost halved during the weekend with respect to workdays. Also, length of stay for patients admitted with hot gallbladder during the weekend is significantly longer than for those admitted during workdays. Surgical operations for small bowel obstruction (SBO) were more often performed by open surgery, but the percentage of those having a laparoscopic operation was higher during the weekend. Similarly, conversion rate was significantly higher during weekends (17.9% vs. 2.4%, *p* = 0.046). Conversion rate was significantly higher during the weekend also for the operations for complicated colorectal cancer (18.2% vs. 0, *p* = 0.031). Patients with complicated inguinal hernia were almost always treated with open surgery both during weekends and weekdays, but the rate of those operated on by laparoscopy was much higher during weekend (4.2% vs. 0.5%).

## Discussion

The so-called weekend effect is a much-studied but yet to be demonstrated and clarified alleged effect claiming that patients admitted and treated during weekends may have different outcomes with respect to those admitted during normal workdays. Most published studies using mortality as primary endpoint gave contrasting evidence [5–8]. An increased weekend mortality was demonstrated for laparotomy, adhesiolysis, colectomy and small bowel resection, but this was due to patients being in poorer conditions and considered more urgent than those treated during workdays [9]. On the contrary, an analysis of the UK National Emergency Laparotomy Audit (NELA) database revealed that quality of care and outcomes for emergency laparotomies did not differ significantly between weekdays and weekends [4].

We decided not to use mortality as endpoint but analysed some of the factors that can potentially impact on mortality and morbidity during weekends. In fact, we wondered whether there was any difference in diagnosis and therapeutical strategy between workdays and weekends.

Moreover, our secondary aim was to verify whether the first wave of COVID-19 pandemic, and consequent lockdown, impacted in any way with the eventual differences of admissions and treatments between workdays and weekends.

We could not demonstrate any significant difference of emergency surgical admissions during weekend as compared to workdays. This is quite interesting as we would have expected that some pathologies, such as pancreatitis, could be more frequent during weekends for cultural reasons (binge drinking, partying, etc.). This has not been demonstrated, but there is an evident trend for “hot gallbladders”, whose incidence tends to increase on Monday, probably associated with a less than healthy diet during weekends. It is possible, but not demonstrated, that the lockdown effect in 2020 might also have played a role. However, these differences were not significant, and it is not unlikely that the profile of admissions for hot gallbladder during the week is only a random effect.

Similarly, we could not demonstrate any difference in the distribution of ASA classes and frailty scores; that is, we could not confirm that patients admitted during weekends are more frail or comorbid. However, a significant difference has been found for both weekdays and weekends in the rate of frail versus non-frail patients admitted into the participating surgical units during the COVID first wave versus non-COVID period, with more frail patients admitted during the COVID period (2020). This finding has already been highlighted and discussed in another paper [2].

Interestingly, the modalities of treatment changed significantly during the weekend, probably due to local availability or reduced adherence to guidance and protocols. In fact, apparently a greater percentage of patients are treated surgically during weekends with respect to working days. This may reflect a more severe acute presentation of those patients, but also the lack of availability of alternative treatments such as interventional radiology or endoscopy.

Among the patients treated with surgery, most have been operated on by open surgery and this percentage did not change during the weekend with respect to working days (57.1% vs. 57.6%), but for some reasons laparoscopic-to-open conversion triples during weekends. This may be related to more advanced presentations during weekends but also to the reduced availability of skilled laparoscopic surgeons in emergency during weekends. It is also possible that organisation factors played a role, with the operating surgeons during the weekend trying to reduce the burden on the already reduced surgical staff by avoiding long and tedious laparoscopic operations.

It is more difficult to explain why this difference was more significant on working days during the COVID-19 pandemic, when there was no conversion in 2020 with respect to 2019. There is the possibility that official guidelines during COVID suggesting the avoidance of laparoscopy as much as possible to reduce the risk of viral transmission through the surgical smoke may have suggested a more selective application of this technique to the easiest cases.

Although length of stay was longer for patients admitted during weekends, their readmission rate is significantly lower. To try to clarify this finding, we performed a regression analysis and a Pearson’s correlation analysis on the entire series to confirm the initial idea that longer stay would be associated with reduced risk of readmission. Surprisingly, both analyses showed that there is a direct correlation (and not inverse as we expected) between length of stay and risk of readmission, probably since patients in poorer general conditions had a longer stay and a higher risk of long-term complications causing readmission. However, an in-depth analysis of this aspect is beyond the scope of this work and may require a wider collection of data.

Zapf et al. found a direct correlation between weekend admission and length of stay, in particular for hot gallbladders [10]. Our subgroup analysis confirmed this finding. This may be possibly explained by the fact that some surgeons (and some units) are not particularly keen to embark into potentially difficult cholecystectomies during the weekend and prefer to postpone the difficult operations to workdays, when subspecialist expertise may be available.

In actual facts, the treatment of acute cholecystitis may be extremely tricky and may need a multidisciplinary approach. Our analysis showed that the interventional treatment for acute cholecystitis (cholecystostomy) may not be widely available during the weekends, hence the reduced percentage of those who benefit of it.

Interestingly, it seems that the laparoscopic approach was used more often during weekend for complicated inguinal hernias and small bowel obstruction. This finding contrasts with the alleged difficulties in performing emergency laparoscopic surgery off-hours and during weekends and represents a very good sign of maturity and flexibility of European health systems. It may be explained, possibly, by the reduced pressure of elective surgery, and the consequently more relaxed environment, on Saturdays and Sundays.

Strengths of this study are its multicentric nature and its large sample. This allowed us to get a reliable snapshot on the differences in emergency surgical admissions and treatments in Europe during weekends versus weekdays. Limitations are its retrospective nature and possibly the imbalanced distribution of participating units, most of them being from Italy.

## Conclusions

Our analysis demonstrated that (1) there is no significant difference in the distribution of emergency diagnoses between workdays and weekend, other than for hot gallbladders, (2) there is no difference in the rate of frail and comorbid patients admitted during workdays with respect to weekend, (3) the first wave of the COVID-19 pandemic did not impact on the weekdays versus weekend relation, (4) the approach to surgical emergencies changes significantly during the week end, probably due to different available skillset and therapeutic capacity, in particular for hot gallbladders, and this may prolong the length of stay and reduce the turnover. This may or may not lead to different outcomes in terms of mortality and morbidity, but managers and policy-makers should be aware of this minimal but significant discrepancy to be able to reshape the emergency surgical services to meet the need of the population with the same level of healthcare 7 days a week.

## Data Availability

The dataset generated and analysed during the current study is available from the corresponding author upon reasonable request.

## Appendix: The CovidICE-International Collaborative

FRANCE

- Paris: Lo Dico R (PLI)

ITALY

- Acireale: Stracqualursi A (PLI), Russo G, D'Errico S
- Andria: Cianci P (PLI), Restini E, Scialandrone G
- Ascoli Piceno: Guercioni G (PLI)
- Bari: Martinez G (PLI), Pezzolla A, Altomare DF, Picciariello A, Trigiante G, Dibra R, Papagni V
- Biella: Righetti C (PLI), Polastri R
- Brescia: Andreuccetti J (PLI), Pignata G, D'Alessio R, Arici E, Canfora I
- Cagliari: Cillara N (PLI), Deserra A, Sechi R
- Castellammare di Stabia: Bianco F (PLI), Gili S, Cappiello A, Incollingo P
- Cattinara: Biloslavo A (PLI), Bellio G, Germani P, De Manzini N
- Cernusco sul Naviglio e Vaprio d'Adda: Buiatti M (PLI), Paladino FP
- Cuneo: Sasia D (PLI), Borghi F, Testa V, Giraudo G, Allisiardi F, Giuffrida MC
- Desio: Gerosa M (PLI), Fogliati A, Maggioni D
- Ferrara: Fabbri N (PLI), Feo CV, Bianchini E, Panzini I
- Foggia (Chirurgia Generale Ospedaliera): Lizzi V (PLI), Tricarico FG, Di Gioia G, Melino R
- Foggia (Chirurgia Generale Universitaria): Tartaglia N (PLI), Ambrosi A, Pavone G, Pacilli M, Vovola F
- Genova: Belli F (PLI), Barberis A, Azzinnaro A
- Grosseto: Coratti A (PLI), Benigni R
- La Spezia: Berti S (PLI), Saracco M, Gennai A, Dova L
- Manerbio: Farfaglia R (PLI), Pata G, Arizzi V, Pandolfo G
- Milano (Fatebenefratelli-Sacco): Frontali A (PLI), Danelli P, Ferrario L, Guerci C
- Milano (Santi Paolo e Carlo): Mariani NM (PLI), Pisani Ceretti A, Nicastro V, Opocher E
- Modena: Gozzo D (PLI), Casoni Pattacini G
- Napoli: Castriconi M (PLI), Amendola A, Gaudiello M, Palomba G
- Parma: Catena F (PLI), Petracca GL, Perrone G, Giuffrida M
- Peschiera del Garda: Moretto G (PLI), Impellizzeri H, Casaril A
- Piacenza: Filosa M (PLI), Caizzone A, Agrusti S, Cattaneo GM, Capelli P
- Pinerolo: Muratore A (PLI), Calabrò M, Pipitore Federico N, Cuzzola B, Danna R, Murgese A
- Pisa: Coccolini F (PLI), Pieroni E, Chiarugi M, Tartaglia D
- Pistoia: Giannessi S (PLI), Somigli R, Trafeli M, Fedi M, De Vincenti R
- Ravenna: Guariniello A (PLI)
- Roma (PTV): Grande M (PLI), Bagaglini G, Pirozzi B, Guida AM, Ingallinella S, Don CP, Siragusa L, Capone O, Cerbo D
- Roma (S.Giovanni): Santoro E (PLI), Pende V, Fassari A
- Roma (Sapienza): Mingoli A (PLI), Brachini G, Cirillo B, Zambon M, Cicerchia P, Meneghini S, Sapienza P, Puzzovio A, La Torre F
- Roma (UCSC): Fransvea P (PLI), Di Grezia M, Sganga G
- Salerno: Armellino MF (PLI), Ioia G, Rampone B, Della Corte M
- Sondrio: Fleres F (PLI), Clarizia G, Bordoni P, Spolini A, Franzini M, Grechi A
- Torino: Suppo M (PLI), Bono D, Scaglione D
- Vimercate: Cotsoglou C (PLI), Paleini S, Chierici AP
- Zingonia: Uccelli M (PLI), Olmi S, Cesana G

PORTUGAL

- Tras-os-Montes e Alto Douro: Tenreiro N (PLI), Marcal A, Martins D, Leal C, Vieira B

SPAIN

- Galdakao: Ugarte-Sierra B (PLI), Vincene-Rodriguez I, Duran-Ballesteros M, Sanz-Larrainzar A, Ibanez-Aguirre FJ
- Saragoza: Yanez-Benites C (PLI), Talal I, Blas JL

UNITED KINGDOM

- Edinburgh: Garau R (PLI), Clark-Stuart S, Wallace A, Di Carlo A, Wisnia E, Ehsan K, Beck-Sanders K, Godson E, Campbell P
- Oxford: Tebala GD (SC), Bignell M (PI), Bond-Smith G, Lewis C, Ahmad R, Ali R, Aswani SS, Barza A, Carrillo C, Dawani A, Dey A, Elserafy A, Gaspar D, Lazzareschi L, Patel M, Shabana A, Shams M, Shams O, Slack Z

PI: Principal Investigator
PLI: Principal Local Investigator
SC: Study Coordinator

## Abbreviations

ACOI: Associazione dei Chirurghi Ospedalieri Italiani (Association of Italian Hospital Surgeons)
ANOVA: Analysis of Variance
ASA: American Society of Anaesthesiologists
CRC: Colorectal cancer
NELA: National Emergency Laparotomy Audit
PI: Principal Investigator
PLI: Principal Local Investigator
SBO: Small bowel obstruction
SC: Study Coordinator
STROBE: Strengthening the Reporting of Observational studies in Epidemiology
WSES: World Society of Emergency Surgery
